# Probability Distortion Depends on Choice Sequence in Rhesus Monkeys

**DOI:** 10.1523/JNEUROSCI.1454-18.2018

**Published:** 2019-04-10

**Authors:** Simone Ferrari-Toniolo, Philipe M. Bujold, Wolfram Schultz

**Affiliations:** Department of Physiology, Development and Neuroscience, University of Cambridge, Cambridge CB2 3DY, United Kingdom

**Keywords:** choice, decision making, primates, reward, risk, utility

## Abstract

Humans and other primates share many decision biases, among them our subjective distortion of objective probabilities. When making choices between uncertain rewards we typically treat probabilities nonlinearly: overvaluing low probabilities of reward and undervaluing high ones. A growing body of evidence, however, points to a more flexible pattern of distortion than the classical inverse-S one, highlighting the effect of experimental conditions in shifting the weight assigned to probabilities, such as task feedback, learning, and attention. Here we investigated the role of sequence structure (the order in which gambles are presented in a choice task) in shaping the probability distortion patterns of rhesus macaques: we presented 2 male monkeys with binary choice sequences of MIXED or REPEATED gambles against safe rewards. Parametric modeling revealed that choices in each sequence type were guided by significantly different patterns of probability distortion: whereas we elicited the classical inverse-S-shaped probability distortion in pseudorandomly MIXED trial sequences of gamble-safe choices, we found the opposite pattern consisting of S-shaped distortion, with REPEATED sequences. We extended these results to binary choices between two gambles, without a safe option, and confirmed the unique influence of the sequence structure in which the animals make choices. Finally, we showed that the value of gambles experienced in the past had a significant impact on the subjective value of future ones, shaping probability distortion on a trial-by-trial basis. Together, our results suggest that differences in choice sequence are sufficient to reverse the direction of probability distortion.

**SIGNIFICANCE STATEMENT** Our lives are peppered with uncertain, probabilistic choices. Recent studies showed how such probabilities are subjectively distorted. In the present study, we show that probability distortions in macaque monkeys differ significantly between sequences in which single gambles are repeated (S-shaped distortion), as opposed to being pseudorandomly intermixed with other gambles (inverse-S-shaped distortion). Our findings challenge the idea of fixed probability distortions resulting from inflexible computations, and points to a more instantaneous evaluation of probabilistic information. Past trial outcomes appeared to drive the “gap” between probability distortions in different conditions. Our data suggest that, as in most adaptive systems, probability values are slowly but constantly updated from prior experience, driving measures of probability distortion to either side of the S/inverse-S debate.

## Introduction

Choices between uncertain rewards require decision-makers to evaluate each option along multiple dimensions. At the very least, a decision-maker needs to simultaneously consider the reward's quantity and probability of occurrence if he is to evaluate its attractiveness in relation to other choice prospects. The von Neumann and Morgenstern Expected Utility (EU) theory was the first axiomatic model of rational behavior capable of describing people's choices in these situations (von Neumann and Morgenstern, 1944). EU theory rigorously introduced the concept of utility as a representation of a decision-maker's subjective value for an objective reward quantity. Through the metric of utility, EU theory was able to describe different risk attitudes, such as the risk-seeking behavior of a gambler or the risk aversion of an insurance buyer; it was, however, soon challenged by the various experimental results of behavioral economics (Weber and Camerer, 1987; for review, see Machina, 1987; Starmer, 2000). Attempts to resolve some of these challenges led to the development of several generalized expected utility theories, many of which (notably prospect theory, rank-dependent utility theory, and cumulative prospect theory) incorporated the concept of probability distortion (Kahneman and Tversky, 1979; Quiggin, 1982; Tversky and Kahneman, 1992). While maintaining the nonlinear relationship between subjective utility and objective reward magnitudes, these theories made use of subjective probability weightings, or probability distortions, to account for the idea that reward probabilities were also treated nonlinearly during choice.

Experimental measures of probability distortion in humans and monkeys typically show that, whereas small probabilities tend to be overweighted by decision-makers, large probabilities are instead underweighted (Kahneman and Tversky, 1979; Gonzalez and Wu, 1999; Stauffer et al., 2015). There is, however, dramatic variation in this pattern of distortion across both different subjects (Gonzalez and Wu, 1999; Bruhin et al., 2010; Burke et al., 2018) and between different task contexts (Hertwig et al., 2004; Wu et al., 2009; Farashahi et al., 2018). While the causes of such variability have yet to be identified, differences in probability distortions could relate to the way in which probability information is presented to decision-makers (Hertwig et al., 2004), or the way in which probability knowledge is acquired and stored by the decision-maker (Camilleri and Newell, 2013). Some studies suggested that prospect theory might altogether be incapable of explaining differences in risk attitudes across these contexts (Kellen et al., 2016).

Here we investigated the role of choice context, specifically sequence structure, as a possible source of probability distortion variability in rhesus macaques, animals known to show quantifiable and reproducible probability distortions (Stauffer et al., 2015). To achieve this, we first measured the certainty equivalents (CEs) of specific gambles, defined as the amount of reward for which the animal was choice-indifferent with regards to said gambles; the CE therefore indicated the subjective value of the gamble in the “currency” of the safe reward. We then simultaneously estimated the contributions of utility and probability distortion to these subjective values, allowing us to model the shape of the monkeys' probability distortion independently from utility.

We used this technique to investigate the possible influence of trial sequence structure on the shape of the probability distortion in two different task situations: randomly intermixing the trials required for the CE measurements of all gambles, or determining the CEs of different gambles via separate blocks of trials. We performed an out-of-sample test to validate and extend the results of our main task, and investigated the contribution of trial history as a possible correlate of probability distortion variance. Our data showed that a change in the presentation order of probability information indeed altered the observed probability distortion pattern, inducing a reversal in probability distortion shape.

## Materials and Methods

### 

#### 

##### Animals and experimental setup.

Two male rhesus macaques (*Macaca mulatta*) were used in this study (11.2 and 13.2 kg). During experiments, the monkey sat in a primate chair (Crist Instruments) and made choices between two rewarding stimuli presented on a computer monitor positioned 30 cm in front of them. The animals reported their choices between options with a left-right motion joystick (Biotronix Workshop). Joystick position and task event times were sampled and stored at 1 kHz on a Windows 7 computer running custom-made software written in MATLAB (The MathWorks) using Psychtoolbox (version 3.0.11). All experimental protocols were assessed and approved by the Home Office of the United Kingdom.

##### Experimental design.

We trained the monkeys to associate visual stimuli with specific juice rewards that varied along two dimensions: the quantity of juice delivered (reward magnitude, *m*) and the delivery probability of the reward (reward probability, *p*). To capture both dimensions descriptively, the visual stimuli consisted of a horizontal bar or of a pair of horizontal bars framed between two vertical lines. The vertical position of the horizontal bars signaled the magnitude of juice delivered; the width of the bar signaled the probability of their delivery from no bar (no reward) to touching the frame on both side (certain reward). To ensure that the bar's edge position relative to the frame was not used as a cue for the gamble's mathematical expected value (EV; i.e., the product of *m* and *p*), the bars were randomly shifted horizontally on each trial. This guaranteed that magnitude and probability information were independently presented and used to make choices. Multiple partial bars found between the vertical frames signaled gambles between “risky” rewards, whereas a singular, full-width horizontal bar signaled a safe, riskless reward. Across all trials, monkeys experienced rewards ranging from 0 ml to 0.5 ml in 0.05 ml increments, and gamble probabilities varying between 0.1 and 1 in decimal increments (0.1).

The animals learned to associate rewards and magnitudes with the visual stimuli schema through >10,000 single-outcome, or imperative, trials. In these trials, only one option was presented on either side of the screen. To obtain the cued reward, the animals were required to select the side on which the reward was presented. All reward options were repeated on both the left and right sides of the computer screen, alternating pseudorandomly to control for any side preference.

Following imperative training, we presented the animals with a binary choice paradigm where they had to choose one of two reward options presented simultaneously. Most binary choice trials pitted a safe reward against a gamble. All gambles consisted of two probabilistic rewards: the monkey could either get a fixed 0.5 ml of juice with probability *p*, or 0 ml of juice with probability 1 − *p*. Safe options varied in terms of reward magnitude only. In separate sets of trials, we presented the animals with choices between two gambles with two outcomes each (possible outcomes: 0, 0.25, 0.5 ml). In these trials, one of the gambles could have two non-zero outcomes (0.25 and 0.5 ml). In all cases, reward was delivered probabilistically, matching the probabilities cued by each stimulus.

**Figure 1.**
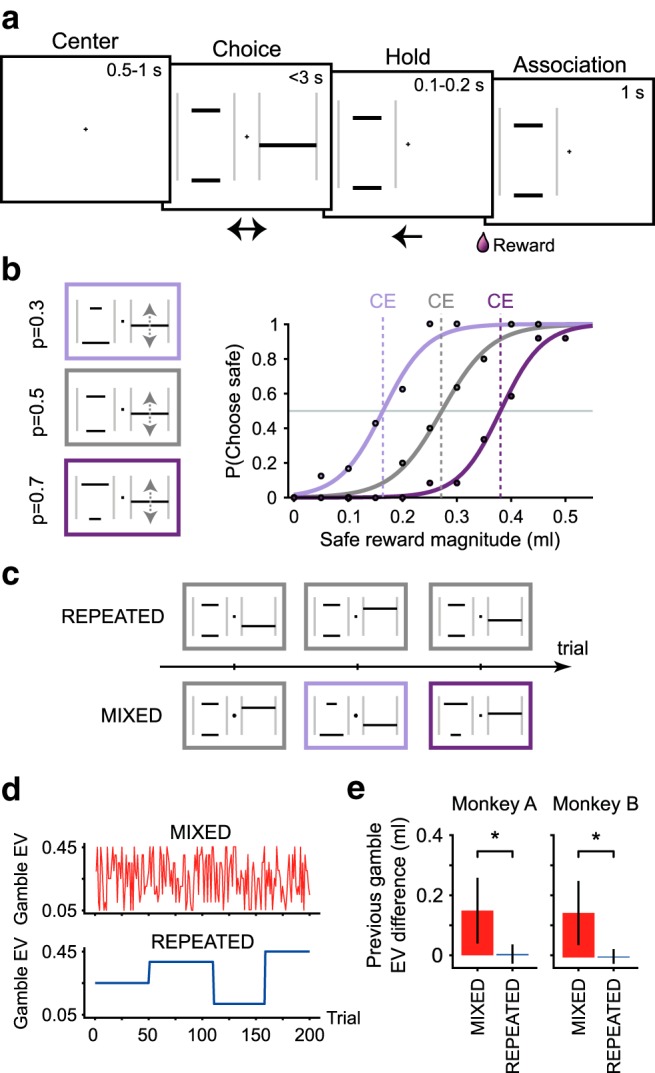
Experimental design. ***a***, Trial sequence. Each trial started with the monkey moving a white cursor, through left/right arm movements with a joystick, to the center of the screen. After 0.5–1 s (center holding), two cues appeared indicating the two offered options (choice period): possible reward magnitudes and probabilities were indicated by the vertical position and width of a horizontal bar, respectively. A single horizontal bar represented a sure reward. Two bars represented a gamble with two possible outcomes. The monkey moved the cursor to the side of the preferred option, within 2 s. After 0.1–0.2 s (holding time), the juice reward was delivered according to the chosen option's reward magnitude and probability. A further 1 s (association period) followed to reinforce the association between chosen cue and reward. ***b***, Psychometric elicitation of CEs. Left, Three example gambles with different reward probabilities (*p* = 0.3, *p* = 0.5, *p* = 0.7) paired with varying safe magnitudes to elicit each gamble's CE. Right, Each point represents the probability of choosing the safe option in choices between a fixed gamble (identified by the color) and a varying safe magnitude (horizontal axis). Lines are psychometric curves obtained by fitting a softmax function to the choice ratios. Each line is associated with one specific gamble and identifies its CE as the magnitude corresponding to a choice ratio of 0.5 (vertical dashed line). ***c***, Task conditions. The CEs were elicited using two sequence structures: in the MIXED condition, different gambles and different safe options were randomly intermixed; whereas in the REPEATED condition, the CE measurement for one gamble was completed before presenting a different gamble. ***d***, Temporal sequence of the presented gamble EV in the two elicitation conditions for one sample session (first 200 trials). The trial-by-trial variation of the gamble EV highlights the difference in sequence structure between MIXED (red) and REPEATED (blue) conditions. ***e***, Variability of gamble EV across consecutive trials. Absolute value of the gamble EV difference (mean ± SEM) between two consecutive trials, showing the main distinction between the two elicitation sequences: the previous trials' gamble EV was consistently different from the current one in the MIXED condition, whereas it stayed constant in the REPEATED condition. *Significant difference between conditions (*t* test).

Trials began with a white cross at the center of a black screen, followed by the appearance of a joystick-driven cursor. The cursor had to be moved to the center cross in order for a trial to begin. After successfully maintaining the cursor on the central cross for 0.5–1 s, two visual option cues appeared left and right of the central cross (see Fig. 1*a*). In the case of imperative trials, only one option appeared while the other side remained dark. The animal had 3 s to convey his decision by moving the joystick to the selected side, after which the unselected option would disappear. The animal's response time (RT; i.e., the time interval between the cues appearance and the beginning of the joystick movement) was collected for individual trials. Reward delivery occurred after the holding time (0.1–0.2 s), and the selected option lingered on the screen for 1 s postreward delivery to reinforce stimulus–reward associations with visual feedback. A variable intertrial period of 1–1.5 s (blank screen) led to the next trial onset. Unsuccessful central hold, side selection hold, or trials where no choices were made resulted in a 6 s timeout for the animal, after which the trial would be repeated.

##### Psychometric elicitation of CEs.

The likelihood of a monkey choosing a specific, individual gamble over different safe options was assessed through the binary choice paradigm (see Fig. 1*b*). The resulting choice ratios were then used to fit a logistic sigmoid function, or psychometric curve, to estimate choice likelihoods for every possible safe-gamble pairing within the tested reward range as follows:

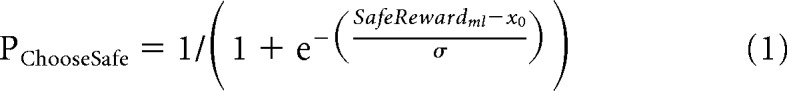
 These psychometric curves captured the likelihood of choosing a safe option over a gamble through two free parameters: x_0_, measuring the *x* position of the curve's inflection point, and σ, the function's temperature parameter, reflecting the steepness of the curve. Importantly, only sequences that contained choices between a gamble and a minimum of three different safe options (repeated at least 4 times) were used in the analysis.

The point of choice indifference between gamble and safe options, corresponding to the inflection point x_0_ of the resulting model, represented a gamble's CE: the certain safe reward that was of equal subjective value to the gamble. CEs could then be used to categorize behavior. Gambles where the CEs were of greater value than the predicted EV signaled risk-seeking behavior for that gamble's probability value. Gambles with CEs lower than their EVs indicated risk-averse behavior for that option. For cases where CEs were equal to EVs, the animals were seen as being risk-neutral.

To explore the role of task structure on the variability of one's probability distortion pattern, we measured CEs in one of two elicitation conditions: MIXED or REPEATED trial sequences (see Fig. 1*c–e*). In the case of MIXED sequences, multiple CEs were elicited through single blocks of randomized choice trials involving different gambles and safe options. Such blocks were repeated until each gamble-safe pair had been presented a minimum of 4 times each. In the case of REPEATED sequences, CEs were elicited using blocks of trials that contained a unique gamble. These REPEATED trial blocks pitted multiple safe options against a single gamble for the elicitation sequence. Other than these sequence designs, everything from visual cues to timescales was identical. The only difference between elicitation conditions was the number of different probabilities of reward (gambles) experienced within a trial block. Testing for each elicitation condition was done consecutively over multiple days, with each monkey receiving imperative training before their daily elicitation sessions. We collected on average 172.95 ± 20.24 (SEM) trials per daily session over 56 sessions for Monkey A (22 REPEATED and 34 MIXED sessions, in consecutive days), and 414.63 ± 27.87 trials over 59 sessions for Monkey B (31 REPEATED and 28 MIXED sessions, in consecutive days).

##### Analysis of behavioral data.

All data were collected, stored, and analyzed using custom MATLAB and Python (SciPy 1.1.0) (Oliphant, 2007) software. Analyses were run on trial-by-trial choice data, and on the CEs elicited psychometrically from these trial-by-trial choices. The data were stored and analyzed separately for the 2 animals.

Before any comparative analyses, the use of visual stimuli to guide the monkeys' decision behavior was verified through analyzing all CE elicitation trials (excluding error trials where the animals made no choices) in a logistic regression model as follows:

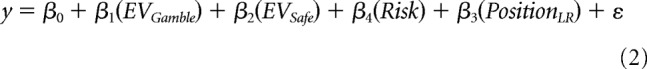
 The dependent variable took a value of *y* = 1 if the gamble was chosen and *y* = 0 if the safe option was chosen instead. As had been previously done (Stauffer et al., 2015), we fitted four independent variables: option values (*EV_gamble_*, *EV_safe_*) were defined as the EVs of gamble and safe rewards; gamble position (*Position_LR_*) as 0 for left, 1 for right screen side; and the outcome's risk value (*Risk*) was defined as $\sqrt{p*\left(1-p\right)}$, a proportional representation of probabilistic variance. We fitted individual testing days separately, fully standardizing the β coefficients and then testing for statistical significance (one-sample *t* test, *p* < 0.05) to identify relevant decision variables. Positive regression coefficients indicated an increase in the likelihood of choosing a gamble over a safe option with increasing independent variable value; negative regression coefficients indicated a decrease in the likelihood of choosing the gamble.

Once the use of onscreen stimuli to guide choices had been confirmed, CEs were measured using the aforementioned psychometric fit. CEs gathered in the MIXED condition were compared with CEs gathered under the REPEATED condition using a two-factor ANOVA with gamble probability and elicitation condition as main factors. The ANOVA also captured any interaction between the two factors, highlighting any condition effects present at a sequence level.

We used trial-by-trial choices to parametrically model the respective effects of utility and probability distortion on single choices, and more generally, on the subjective value of gambles (CEs). For each daily testing session, we simultaneously estimated both the utility and probability distortion functions from within a standard discrete choice model. Functional parameters that best described choices between gamble-safe pairs were elicited in this way, capturing the individual effects of nonlinear utility and probability distortion. The model ran on trial-by-trial choice data, with data binned into several sets containing one gamble and all safe options presented against it on the day (CE elicitation sequence). The discrete choice (softmax) function returned the probability of choosing the gamble option based on the subjective value of both the gamble (*V_G_*) and the safe reward presented (*V_S_*).

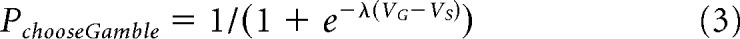
 The softmax parameter, λ, defined the likeliness of choosing the better prospect; each option's value (*V*) was defined according to prospect theory (Kahneman and Tversky, 1979), as the product of utility (*u*) and probability distortion (*w*) outputs as follows:

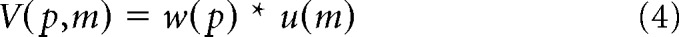
 Utility was modeled through a power function as follows:

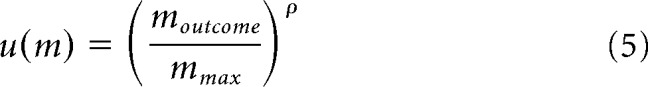
 where ρ > 1 captured risk-seeking choice behavior, ρ < 1 captured risk-averse choice behavior (ρ < 1), and ρ = 0 implied risk neutrality (Hsu et al., 2009). Magnitude values were divided by 0.5 ml (*m_max_*), such that the maximal reward a monkey could get was anchored at 1 unit of utility.

We compared four functional models of probability distortion in an attempt to best capture changes in probability distortion across conditions. Of these classical models, two had a single fitting parameter: the one-parameter Prelec function (Eq. 6, *Prelec-1*, parameter: α) and the Kahneman and Tversky probability weighting function (Eq. 7, *Tversky,* parameter: ε); the others had two fitting parameters: the two-parameter Prelec function (Eq. 8, *Prelec-2*, parameters: α, β) and the Gonzalez and Wu log-odds model (Eq. 9, *Gonzalez*, parameters: γ, δ). Formally:

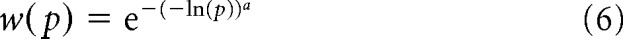


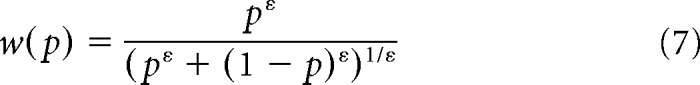


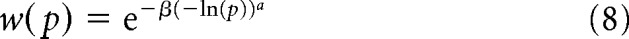


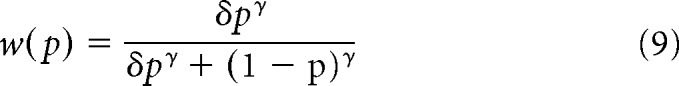
 Using a maximum likelihood estimation (MLE) method, we simultaneously estimated the functional parameters (Θ) from the experimental data. We defined the log-likelihood function as follows:

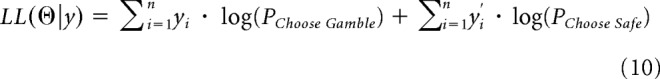
 The log-likelihood function was defined on all trials in a session (*n*), the trial number (*i*), and the choice outcome variable for the gambles and safe options (*y* and *y′*, respectively). The outcome variables took a value of 1 if their respective option was chosen; 0 otherwise. We used an unconstrained Nelder–Mead search algorithm (MATLAB: fminsearch) to compute the functional parameters that minimized the negative log-likelihood (−*LL*). This MLE approach allowed for the simultaneous estimation of the model's free parameters, placing no constraints on their values (Abdellaoui, 2000; Pelé et al., 2014; Stauffer et al., 2015).

The algorithm identified the best fitting softmax, utility, and probability distortion parameters with respect to each monkey's daily choices in CE elicitation sequences. Four complete models were parametrized, accounting for the different probability distortion functions investigated. From these, we calculated the Bayesian Information Criterion (BIC) to pinpoint the probability distortion function most reliable in capturing behavior. Four sets of parameters and their BIC were estimated for every testing day, independently for each model. We selected a single model for further analysis, based on the flexibility of the functional model, its comparative BIC score (one-factor ANOVA with repeated measures, Greenhouse-Geisser–corrected *p* values: *pGGc*), and the deviance between the model's predicted CEs and the experimental ones (one-factor ANOVA with repeated measures, Greenhouse-Geisser–corrected *p* values) (Greenhouse and Geisser, 1959).

We further validated the parameter estimation procedure by running 10 simulated choice datasets within the fitting algorithm. Datasets used for testing were generated by fixing the utility parameter (ρ) and varying the probability distortion parameter (α), or vice versa. The softmax temperature parameter was kept constant (λ = 10), as we specifically wanted to test the robustness of the estimation procedure in relation to variability in the utility and probability parameters. These fixed models were used to simulate individual trial choices. We simulated 6 trials for every gamble-safe pair (safe magnitude levels: 0–0.5 ml in steps of 0.05 ml). Five datasets varied in terms of utility (ρ = 0.20, 0.50, 1.00, 1.50, 3.00), and five in terms of probability distortion (α = 0.33, 0.67, 1.00, 1.50, 3.00). We measured estimation accuracy as the 95% CI on estimated parameters from Monte Carlo simulations on the parameter-derived datasets.

The final estimated parameters were first log-transformed to account for the asymmetric distribution of the utility and probability distortion parameters (ranging from 0 to ∞, with a value of 1 defining the linear case). We then compared the parameter estimates via one-way MANOVA with elicitation condition as main factor. From this multivariate analysis, we identified any significant effect of individual decision functions while recognizing the collective role all three parameters in capturing risk preference. More specifically, the MANOVA identified which model function parameters (choice softmax, utility, or probability distortion) differed significantly between CE elicitation conditions.

In the REPEATED condition, the gamble option did not change for long sequences of trials and could, theoretically, be ignored. To test the possibility of an attentional shift toward the safe option in this condition, we defined a model with different weights applied to the two options' values as follows:

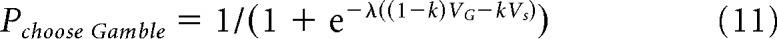
 The weight parameter (*k*) captured the attentional shift toward one option, if significantly >0.5. The options' values (*V_G_*, *V_S_*) were computed, as in the previous model, using the power utility function and the selected probability distortion function (*Prelec-1*).

##### Evaluation of probability distortion in the Marschak–Machina triangle.

We introduced the Marschak–Machina triangle (Marschak, 1950; Machina, 1982) to compare the choice behavior between the MIXED and REPEATED conditions in an out-of-sample test, and to evaluate the theoretical predictions of the discrete choice model vis-à-vis utility and probability distortions.

The Marschak–Machina triangle defines a 2D space where any probabilistic combination of three fixed reward magnitudes *m*_1_ < *m*_2_ < *m*_3_ can be represented (for details, see Results). The *x* and *y* axes correspond to the probability of obtaining the lowest (*p*_1_) reward *m*_1_ and the highest (*p*_3_) reward m_3_, respectively. The probability of the middle magnitude is not explicitly represented in the diagram, but it can be readily obtained as *p*_2_ = 1 − (*p*_1_ + *p*_3_). Points on the horizontal axis therefore correspond to gambles with outcomes *m*_1_ and *m*_2_, whereas points on the vertical axis identify gambles with *m*_2_ and *m*_3_ as possible outcomes; the hypotenuse comprises all gambles containing outcomes *m*_1_ and *m*_3_ only. In our experiment, we set the fixed magnitude levels to *m*_1_ = 0 ml, *m*_2_ = 0.25 ml, and *m*_3_ = 0.5 ml.

We characterized Monkey A's behavior within the Marschak–Machina triangle, by defining indifference lines between points on the triangle edges as follows: we presented choices between a fixed gamble (A), defined on one of the axes, and a set of gambles (B_i_) located on the triangle's hypotenuse; by fitting a psychometric curve to the ratio of B_i_ and A choices, we identified the indifference point on the hypotenuse as the probability *p*_3_ corresponding to a choice ratio of 0.5. We then defined an indifference line as the segment connecting the fixed gamble on the axis with its corresponding indifference point. This procedure was repeated for four fixed gambles on the *x* axis (*p*_1_ = 0.2, 0.4, 0.6, 0.8) and for another four fixed gambles on the *y* axis (*p*_3_ = 0.2, 0.4, 0.6, 0.8), resulting in 8 indifference lines.

Such indifference lines characterized points on the triangle edges (two-outcome gambles): they did not represent complete indifference curves within the Marschak–Machina triangle (three-outcome gambles). Nevertheless, the slopes of the indifference lines univocally identified a directional property a monkey's risk preference pattern: a gradual change in the slope (fanning-in or fanning-out) of indifference lines has been extensively used in the economic literature to characterize choice behavior, particularly in relation to the predictions of generalized expected utility theories. This property allowed us to quantify behavioral changes across elicitation conditions and to compare the observed data with predictions from the theoretical economic model.

Crucially, gambles resting on the two axes were never used in the elicitation of CEs, representing an out-of-sample test. As a consequence, the choice behavior observed in the Marschak–Machina triangle could be used as independent validation for our previous results.

We computed the theoretical indifference lines by calculating, for each of the eight fixed gambles defined above, the probability *p*_3_ for which the theoretical subjective value of the fixed gamble was equal to that of the gamble on the hypotenuse. The subjective value of a two-outcome gamble was defined according to cumulative prospect theory as follows:

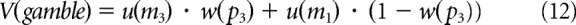
 where *m*_3_ and *m*_1_ represent the magnitude of the highest and lowest outcome, respectively, *p*_3_ the probability of occurrence of the highest outcome, *u* the power utility function, and *w* the *Prelec-1* probability distortion function.

The indifference point was defined as the point on the hypotenuse with subjective value (*u*(*m*_3_) · *w*(*p*_3_)) equal to the subjective value of the fixed gamble (*V*(*gamble*)). Thus, knowing the value of the fixed gamble, one could identify the indifference point as the probability *p*_3_ satisfying the equation *u*(*m*_3_) · *w(p*_3_) = *V*(*gamble*) as follows:

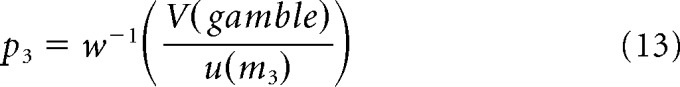
 where *w*^−*1*^ represents the inverse of the probability distortion function: that is, *w*^−*1*^ = *exp(*−*(*−*ln(w))*^*1*/α^*).*

Each daily set of indifference points was elicited after CE elicitation sequences, for both the MIXED and REPEATED CE elicitation sessions. This resulted in two sets of indifference lines, distinctly associated with the REPEATED and MIXED conditions. Both datasets were obtained using intermingled gamble sequences, so any difference in the pattern of indifference lines could only be attributed to the effect of the previous block of trials (i.e., REPEATED or MIXED CE elicitation).

The directional pattern of the indifference lines was characterized by a measure of the “fanning” direction, corresponding to a gradual change in the slopes of indifference lines. When moving from the lower right to the top left corner of the Marschak–Machina triangle, indifference lines decreasing their slope would fan-in, whereas indifference lines increasing their slope would fan-out, much like the structural slats of a folding fan.

A linear regression analysis on the indifference line slopes was used to statistically characterize the fanning pattern. A positive regression coefficient identified fanning-out of the indifference lines, whereas a negative regression coefficient identified fanning-in. It should be noted that the relation between the slopes of the indifference lines, as we defined them, was not expected to be linear, but the linear regression served as a reasonable description of the expected theoretical pattern and was then used to characterize the measured behavior.

To statistically compare the predicted and observed sequence effects on the steepness of the indifference lines, we first calculated the shift of indifference points (change in *p*_3_ value) between the REPEATED and MIXED conditions; we did this for each of the eight indifference lines, for both the measured data and the model's predicted lines. We then performed a correlation analysis on the modeled and measured shifts.

##### Trial history effects.

Because gamble presentation order was the only difference between the MIXED and REPEATED elicitation sequences, we sought to categorize the effects of said order on the subjective distortion of probabilities. Using past gamble EVs as a quantitative measure of past experiences (specific to probabilities) we compared the distribution and use of previous gamble EVs across elicitation condition.

We first compared the variability of consecutive gamble probabilities in both conditions using a two-sample *t* test. We used the absolute value of consecutive gamble EV differences to contrast order in an unsigned manner, as signed differences would amount to 0 in both cases. We then assessed the use of past gamble EVs using the following logistic regression:

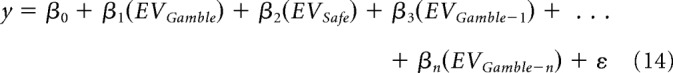
 Again, the dependent variable took a value of *y* = 1 if the gamble was chosen and *y* = 0 if the safe option was chosen instead. The EV of both the current gamble and safe (*EV_gamble_*, *EV_safe_*), as well as the gamble EV of up to 8 trials in the past (*EV*_*gamble*−*n*_), served as independent variables. Trials that did not have a minimum of 8 previous trials, in individual sessions, were removed for this analysis. We again standardized regression coefficients and identified how many past gamble EVs had a significant impact on current choice (one-sample *t* test, *p* < 0.05). Refining the analysis to a singular preceding trial, we investigated the use of a win-stay/lose-shift (WSLS) strategy by the animals. A common strategy for human and nonhuman primates alike, a WSLS choice pattern involves repeating a “winning” choice until it results in a “loss,” after which one would shift and try their luck on another choice option. Because choice options in the CE elicitation sequences involved many different values for both the gamble and the safe options, we instead explored a more relaxed WSLS model as follows:

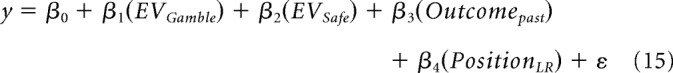
 If the previous choice had been that of a gamble, and that gamble had won (i.e., resulted in a 0.5 ml reward), the third independent variable (*Outcome_past_*) took a value of 1; if the past chosen gamble had instead been unsuccessful, *Outcome_past_* was 0. By including current *EV_Gamble_*, *EV_Safe_*, and *Position_LR_*, we could identify the relative effect of a previous gamble's outcome on current choice. The logistic regression analysis was only applied to trials in which the previous trial's gamble was chosen. A positive regression coefficient for *Outcome_past_* implied a greater likelihood of picking the gamble after a “win”, regardless of its value. A negative coefficient would, instead, capture a decrease in the likelihood of picking the gamble, whatever it may be, after a “loss.”

To compare the performance of this model with the previously defined model (Eq. 2), which did not include the contribution of past trials, we computed the BIC scores of the two models only in trials in which the previous gamble was chosen. After this trial selection, we removed 5 sessions in Monkey A's data, as they had fewer than 4 trials per gamble-safe pair.

To further investigate the effect of past outcomes on the risk patterns, we defined a reinforcement learning model, in which each gamble value was updated, starting from its EV, by adding or removing a fixed amount following a win or a loss, respectively. Formally, choices were evaluated according to the discrete choice model defined earlier (Eq. 2), in which the safe value (*V_S_*) was the certain option's magnitude (linear coding of magnitudes), whereas the gamble value (*V_G_*) was updated on each trial according to the following rule:

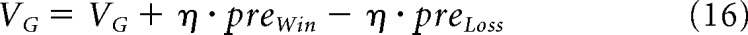
 Where *pre_Win_* and *pre_Loss_* are variables encoding the last trial's outcome: *pre_Win_* = 1 if a gamble was won in the previous trial, 0 otherwise, and vice versa for *pre_Loss_*. The value-updating parameter η represents the amount of “value” (in milliliters) added or removed to the gamble value based on the previous outcome. According to this model, the gamble value was not updated if the safe option had been chosen on the previous trial.

We retrieved the η parameter value using MLE, and used the resulting average value to simulate choices and compute the resulting CEs. The simulation was run on MIXED and REPEATED sequences separately, to compare the effect of a value-updating model on the CEs in the two sequence conditions.

##### Statistical analysis.

We used MATLAB and/or Python for all statistical analyses. Logistic regressions were computed per session, and results were standardized by multiplying each coefficient with the ratio of the corresponding independent variable's SD over the SD of the predicted variable (Menard, 2011). Standardized regression coefficients were tested for statistical significance through one-sample *t* test. Two-factor ANOVA, one-factor MANOVA, linear regression, and *t* test results were considered significant at *p* < 0.05, whereas one-way repeated-measures ANOVAs were Greenhouse–Geisser corrected (degrees of freedom adjustment) to account for sphericity violations (Mauchly's test *p* < 0.05; Greenhouse and Geisser, 1959). *Post hoc* analysis with Bonferroni–Holm correction for multiple comparisons was applied to ANOVA results. Cohen's *d* values were used as a measure of effect sizes. In all analyses of data from single sessions, we reported mean ± SEM across sessions.

## Results

### Design

We tested whether the shape of the probability distortion would be influenced by the order in which probability information is presented in a sequence of decisions.

Once the animals had been extensively trained with the reward-predicting stimuli (>10,000 trials), we presented them with sequences of binary choices between different probabilistic rewards (or gambles) and safe rewards (Fig. 1). We then used the choice ratios to measure the value of gambles relative to certain rewards, pinpointing the certain rewards that were subjectively equivalent to gambles, or a gamble's CE. This procedure revealed the animals' attitude toward risky choices: gamble CEs larger than said gamble's objective EV reflected risk-seeking behavior; risk-aversion was characterized instead by gamble CEs smaller than the gamble's EV.

By simultaneously estimating the individual contributions of utility and probability distortion to these measures of risk attitudes, we could model the shape of the monkeys' probability distortion regardless of the utility function.

### Basic behavioral performance

**Figure 2.**
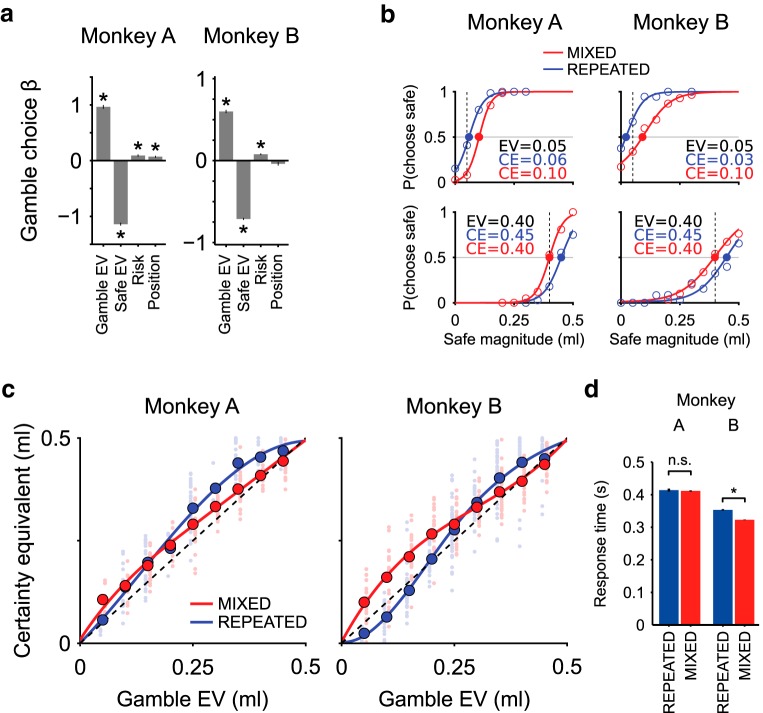
Basic choice behavior and estimation of CEs. ***a***, Logistic regression of choice behavior. Four task variables (gamble EV, safe EV [magnitude], risk variance, gamble position) were used as regressors for the gamble choice. Positive standardized coefficients for gamble EV and risk indicated that monkeys preferred gambles with higher EV to gambles with lower EV, and more risky gambles to less risky ones. Negative coefficient for safe EV confirmed that monkeys preferred higher reward magnitudes to lower ones. The positive position factor for 1 monkey indicated a side bias, which was taken into account by repeating all choice pairs with inverted left-right positions.* Significant regression coefficient (one-sample *t* test). ***b***, Psychometric estimation of CEs. CEs of two example gambles with probabilities 0.1 (top) and 0.8 (bottom), estimated in the two different elicitation sequences: MIXED (red) and REPEATED (blue) sequences. The percentages of safe choices as a function of safe magnitude (circles) were fitted to softmax functions (curves). Vertical lines indicate the gambles EVs (dashed lines). Filled circles represent the CEs. In both monkeys, low probability gambles (top) had a lower CE in the REPEATED condition than in the MIXED condition, where the CEs were consistently higher than the EVs, indicating risk seeking behavior. High probability gambles (bottom) showed the inverse pattern, indicating risk seeking behavior only in the REPEATED condition. ***c***, Pattern of CEs across the two elicitation sequences (MIXED vs REPEATED). Single session CEs (small data points) and average CEs across sessions (large data points) plotted as a function of gamble EV, with cubic spline interpolated curves. The full pattern of CEs shows a smooth transition from low to high probability gambles in terms of CE difference across the two elicitation sequences. For low probability gambles, both monkeys showed higher CEs in the MIXED than in the REPEATED conditions; when increasing gamble probabilities, the CE difference across conditions gradually reduced and inverted, resulting in lower CEs in the MIXED than in the REPEATED condition for high reward probabilities. Single session data points were shifted horizontally (REPEATED condition: left; MIXED condition: right) for visualization purposes. ***d***, Mean RT (± SEM across sessions) in the two elicitation conditions. RTs for Monkey A were similar in the two conditions (RT difference = 3.0 ms, *t*_(9088)_ = −0.59, *p* = 0.56); Monkey B showed faster response in the MIXED condition compared with the REPEATED condition (RT difference = 30.0 ms, *t*_(22233)_ = −15.88, *p* = 1.77 × 10^−56^) (for RT as a function of the options' EV, see Figure 2-1). *Significant RT difference between conditions (two-sample *t* test).

A logistic regression analysis demonstrated that the monkeys used the information from the visual stimuli to guide their decisions on all daily testing sessions (Fig. 2*a*). A positive regression coefficient for gamble EV (one-sample *t* test, Monkey A: *t*_(55)_ = 29.41, *p* = 2.5 × 10^−35^; Monkey B: *t*_(58)_ = 30.16, *p* = 3.9 × 10^−37^) indicated that animals were more likely to choose higher probability gambles than lower probability ones; conversely, the negative coefficient for safe reward EV (Monkey A: *t*_(55)_ = −44.65, *p* = 6.8 × 10^−45^; Monkey B: *t*_(58)_ = −58.61, *p* = 2.6 × 10^−53^) indicated that monkeys chose the safe option more frequently when its value was of higher magnitude. Both animals preferred gambles of higher over lower probabilistic variance (i.e., they preferred gambles that were more uncertain, regardless of the outcome) (positive coefficient for risk; Monkey A: *t*_(55)_ = 4.58, *p* = 2.7 × 10^−5^; Monkey B: *t*_(58)_ = 7.79, *p* = 1.4 × 10^−10^). Monkey A, but not Monkey B, showed a side bias (positive coefficient for the position variable), which was taken into account by balancing the positions of gambles and safe rewards: every option was presented the same number of times on each side of the computer monitor.

10.1523/JNEUROSCI.1454-18.2018.f2-1 Figure 2-1 Response time vs EV. Top: Mean RT (± SEM across sessions) as a function of EV difference between the two presented options (gamble EV - safe magnitude). Choices between options with similar EV produced higher RT. Bottom: Mean RT (± SEM across sessions) as a function of the EV of the chosen option. Faster RTs were associated to higher EV of the chosen option, while slower RTs corresponded to choices where a low EV option was selected. Download Figure 2-1, TIF file

### Estimation of subjective values using different sequence structures

We used a binary choice paradigm to estimate the monkeys' subjective valuation of specific gambles. We measured the choice ratios between different safe rewards and gambles ranging in probabilities from *p* = 0.1 to *p* = 0.9. Fitting a softmax curve to each of these gamble-safe groups allowed us to estimate the CEs corresponding to different gamble probabilities (see Materials and Methods). These CEs served as a measure of subjective value for unique probabilities and provided us with a precise measure of an animal's risk preference over the range of probabilities tested.

We elicited CEs in both monkeys using two different elicitation conditions: MIXED and REPEATED gamble sequences (Fig. 2*b*). In the MIXED condition, we estimated CEs from sequences of binary choices containing several different gambles pitted against safe rewards. All gamble and safe options presented were randomly intermixed, and multiple CEs were estimated from these sequences, one for each gamble. In the REPEATED condition, we elicited CEs from blocks of trials that contained a single, unique gamble versus different safe rewards. In this way, we elicited a unique gamble's CE for each given block. Importantly, the two conditions used the same visual stimuli; any difference between estimated CEs would therefore be due to the elicitation sequence in which CEs were estimated.

We aggregated the daily CEs of individual monkeys, for both conditions, to determine the risk-preference pattern derived from the CEs measured in each elicitation sequence. The risk-preference pattern was therefore directly inferred from the relation between the CEs and the respective EVs, as opposed to being theoretically derived from the shape of utility and probability distortion functions. We found a significant difference between the distribution of CE values elicited in REPEATED versus those elicited in MIXED sequences (two-way ANOVA, factors: gamble probability, elicitation condition). As expected, we found a significant main effect of reward probability on a gamble's CE: higher probability gambles had a higher CE in both animals (Monkey A: *F*_(8,237)_ = 444.12, *p* = 5.2 × 10^−138^; Monkey B: *F*_(8,337)_ = 241.14, *p* = 1.4 × 10^−134^). We also saw a main effect of elicitation conditions (Monkey A: *F*_(1,237)_ = 7.69, *p* = 0.006; Monkey B: *F*_(1,337)_ = 20.21, *p* = 9.6 × 10^−6^), where CEs elicited in the MIXED condition were significantly different from those in the REPEATED condition. Adding to this effect, we observed a significant interaction effect between probability and condition (Monkey A: *F*_(8,237)_ = 7.73, *p* = 3.3 × 10^−9^; Monkey B: *F*_(8,337)_ = 12.56, *p* = 8.5 × 10^−16^), suggesting that the different elicitation sequences had a more complex effect on CE values than a mere monotonic increase or decrease. This effect was readily observable from the condition-specific CE distributions (Fig. 2*c*), where the concave pattern of the MIXED-condition CEs contrasts with the S-shaped distribution of the REPEATED-condition CEs. Analysis of the RTs showed no significant difference across conditions for Monkey A, while monkey B responded faster in the MIXED than in the REPEATED condition (Fig. 2*d*). In general, monkeys showed a consistent RT pattern (Fig. 2-1): shorter RTs when choosing higher EV compared to lower EV options, and longer RTs for smaller EV differences between options.

### Sequence-dependent changes in probability distortion

Because CE elicitation rested on reward options that varied in both magnitude and probability, any risk-preference changes could be attributed to nonlinear utility, probability distortion, or a combination of both. To better understand the role of these decision variables in shaping a gamble's subjective value, we simultaneously estimated the shape of both functions from the monkeys' daily binary choices. Using a standard discrete choice model (Eq. 3), we elicited functional parameters that best explained each animal's choices between gamble-safe choice pairs on individual days, assuming nonlinear utility and probability distortion. The estimation procedure allowed parameters to take on any value, imposing no constraints beyond the functional forms of the discrete choice softmax, probability distortion, and utility curves.

We defined the value of each reward option as the product of its subjective probability and utility, consistent with prospect theory and other modern decision theories (Kahneman and Tversky, 1979; Tversky and Kahneman, 1992). As is traditionally done, we modeled utility through a one-parameter power function. The simple power function accounted well for risk-seeking (ρ > 1), risk-averse (ρ < 1), or risk neutral attitude (ρ = 1) for the range of reward magnitudes. We tested only one model for utility, as magnitude presentations did not differ across conditions. Instead, we sought to optimize our choice model with regards to subjective probability because CE elicitation sequences differed in terms of the order in which gamble probabilities were experienced. We tested four classical models of probability distortion to maximize the reliability of our model in capturing real choices; two of these functions had one free parameter, and the others had two. Finally, we defined cumulative log-likelihood functions for each of these models and estimated the best-fitting parameters for each decision function through MLE (see Materials and Methods).

**Figure 3.**
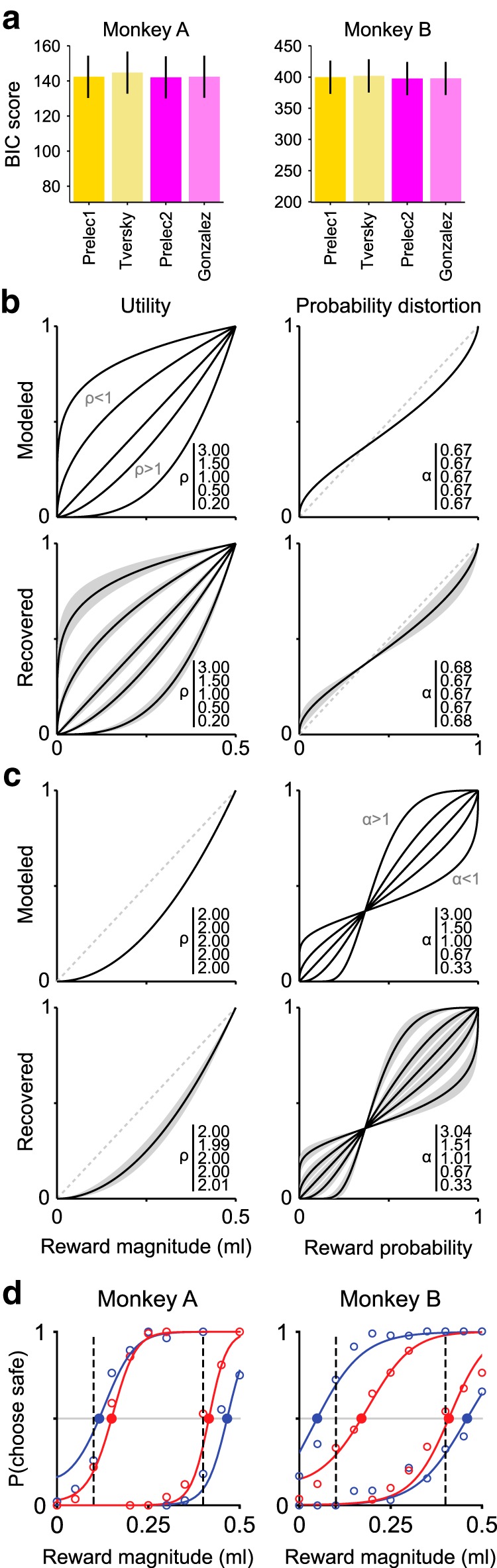
Choice model selection and validation. ***a***, Goodness-of-fit for choice behavior using four models with different probability weighting functions. Bars represent mean BIC values (±SEM) across all sessions (Monkey A: *N* = 56; Monkey B: *N* = 59). BIC scores for daily parametric fits differed significantly across models (one-factor ANOVA with repeated measures, Monkey A: *F*_(3,150)_ = 8.32, *pGGc* = 3.1 × 10^−3^; Monkey B: *F*_(3,174)_ = 13.575, *pGGc* = 5.3 × 10^−08^). Lower BIC values for the Prelec weighting functions (*Prelec-1*, *Prelec-2*) indicate a better fit of the data compared with the one-parameter Tversky or two-parameter *Gonzalez* functions. BIC values for all model pairs, except for *Prelec-1* versus *Prelec-2*, *Prelec-1* versus *Gonzalez*, *Prelec-2* versus *Gonzalez* in Monkey A, and the *Prelec-2* versus *Gonzalez* in Monkey B, were significantly different (*post hoc* analysis, *p* < 0.05) for both monkeys. The sum of squared errors in CE estimation was the lowest in the Prelec models. ***b***, ***c***, Validation of the parameter estimation procedure using the *Prelec-1* probability weighting function. Top, Utility (left) and probability distortion (right) functions used to simulate choices. Bottom, The functions recovered with the MLE procedure. Monte Carlo simulation of choice behavior (using the same number of trials and the same step-size for magnitude and probability as in the measured data: 9 gamble probabilities, 11 safe magnitudes, 6 trials per gamble-safe pair) was repeated 1000 times, producing the 95% CIs on the parameter estimates (gray areas). Varying the utility function parameter (ρ, 0.2–3) while keeping the probability distortion parameter constant (α = 0.67) resulted in an unbiased estimate of the utility shape (***b***). The probability distortion parameter (α), varying from 0.33 to 3 while keeping the utility shape fixed (ρ = 2), was recovered consistently and without bias (***c***). ***d***, Modeled versus measured choice behavior. Comparison of estimated (curves) and measured (circles) percentage of safe choices as a function of safe magnitude, for two example gambles (probabilities 0.2 and 0.8) (for the full dataset, see Figure 3-1). Estimated choice percentages were computed using the discrete choice model with the MLE-recovered parameters (Eq. 3, using the *Prelec-1* probability weighting function). Red and blue points represent estimated CEs. Vertical dashed lines indicate EVs. The estimated psychometric functions closely approximated the measured data points, and differences in estimated CEs across conditions are compatible with the observed data for both low and high probabilities (Fig. 2*b*).

Across all testing sessions, the BIC scores of the Prelec curves were consistently lower than the one-parameter Tversky and lower than the Gonzalez models in at least monkeys (Fig. 3*a*). However, while the two-parameter Prelec had a marginally lower BIC score in both animals, the one-parameter Prelec showed had a marginally lower sum of squared errors between predicted and average experimental CEs (one-factor ANOVA with repeated measures, Monkey A: *F*_(3,144)_ = 6.166, *pGGc* = 5.7 × 10^4^; Monkey B: *F*_(3,168)_ = 3.699, *pGGc* = 1.3 × 10^−2^). We ultimately selected the one-parameter Prelec due to this lower sum of squared errors, lower parameter count, and because of its ease of interpretation: for the curvature parameter α > 1, the function underweighted low probabilities and overweighted high ones, for α < 1, low probabilities were overweighted and high ones were underweighted. With an α = 1, probabilities were treated linearly. Monte Carlo simulations from predefined parameters confirmed the reliability of the MLE method for the selected model: we recovered accurate parameters for both the utility (Fig. 3*b*) and probability distortion (Fig. 3*c*) functions.

10.1523/JNEUROSCI.1454-18.2018.f3-1 Figure 3-1 Modeled vs measured choice behavior. Comparison of estimated (curves) and measured (circles) percentage of safe choices as a function of safe magnitude. Conventions and symbols as in Fig. 3d. Thin lines represent differences between estimated and experimental data percentages, with the horizontal line (at 0.5 on the y axis) corresponding to perfect estimate (difference=0). Download Figure 3-1, TIF file

Having selected the one-parameter Prelec as the best-fitting probability distortion function, we estimated the functional parameters of our choice model (Eq. 3) using the MLE method. The model was able to capture the characteristic pattern of risk attitudes observed in our experimental data: CEs of low probability gambles resulted in larger than the respective EVs in the MIXED condition, whereas CEs of high probability gambles were larger than their EVs in the REPEATED condition (Fig. 3*d*, see Fig. 3-1 for the full dataset), in accordance with the measured behavior (Fig. 2*b*).

**Figure 4.**
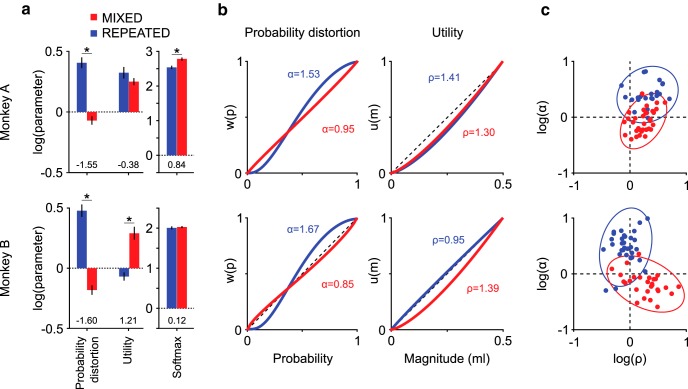
Utility and probability distortion functions in two elicitation conditions. ***a***, Model parameter estimates (mean ± SEM across sessions) in the MIXED (red) and REPEATED (blue) conditions. *Significant differences across conditions (MANOVA). The probability distortion parameter (α) consistently varied across sequence structures in both monkeys: negative log values in the MIXED condition corresponded to inverse S-shaped probability distortion (α < 1), whereas positive log values in the REPEATED condition implied S-shaped probability distortion (α > 1). Numbers below the bars indicate effect sizes (Cohen's *d*). The utility (ρ) and softmax (λ) parameters significantly differed across conditions only for 1 monkey, with a smaller effect size compared with the probability distortion parameter. ***b***, Shapes of the probability distortion function (left) and utility function (right) corresponding to the estimated parameters, displaying the consistent difference in subjective probability evaluation across conditions for both monkeys. ***c***, 2D representation of the utility and probability distortion parameter estimates. Dots represent the simultaneously estimated utility (ρ) and probability distortion (α) parameters for single behavioral sessions, with 95% confidence ellipses.

We compared daily estimated parameters across CE elicitation conditions for utility and probability distortion (Fig. 4*a*). Both animals exhibited convex utility (ρ > 1) in the tested range of 0–0.5 ml accounting for risk-seeking behavior, with linearity only in the case of Monkey B's REPEATED condition. Importantly, probability distortions inverted across elicitation condition. In the MIXED elicitation condition, both animals overweighted low probabilities and underweighted high ones (α > 1), whereas they instead underweighted low probabilities and overweighted high ones within the REPEATED condition (α < 1) (Fig. 4*b*). MANOVA confirmed the impact of the different elicitation sequences on both animals' choice pattern (Monkey A: *F*_(1,54)_ = 24.96, Wilks's λ = 0.41, *p* = 3.85 × 10^−10^, η^2^ = 0.59; Monkey B: *F*_(1,57)_ = 40.78, Wilk's λ = 0.31, *p* = 5.2 × 10^−14^, η^2^ = 0.69) with only the probability distortion parameter (α) consistently different across conditions (Fig. 4*a*,*c*). The change in risk-attitude between the two conditions could therefore, at least in the case of gamble-safe choices, be reduced to a reversal in the probability distortion function.

The REPEATED condition was a much less complex decision situation compared with the MIXED one, theoretically allowing for a simpler choice strategy: it would have been sufficient to evaluate the certain option, ignoring the gamble option in the majority of trials, to make choices. We tested for this possibility by fitting a model with an attentional parameter to the choice data (Eq. 11). We found that there was no significant difference in attention given to the safe compared with the gamble option (the weight parameter was not significantly different from 0.5; Monkey A: *t*_(21)_ = −2.01, *p* = 5.7 × 10^−2^ (*t* test); Monkey B: *t*_(30)_ = −1.25; *p* = 2.2 × 10^−1^), suggesting that both options were fully considered when making choices in the REPEATED condition. Furthermore, shorter RTs in the REPEATED condition, expected if the monkeys ignored the gamble option, were not observed (Fig. 2*d*).

### Reversal of probability distortion in the Marschak–Machina triangle

To extend our findings beyond gamble-safe choices, we characterized the choice behavior of 1 monkey in a different set of gambles using the Marschak–Machina triangle. This diagram was first introduced as a way of “organizing” a series of anomalies observed in risky choices, most notably the common ratio and common consequence effects, which violated the independence axiom of EU theory (Allais, 1953). Several economic theories were developed to explain these apparent paradoxes. Each theory predicted indifference curves with distinctive shapes in the Marschak–Machina triangle, making it an ideal framework to evaluate and compare the alternative theories (Machina, 1982).

**Figure 5.**
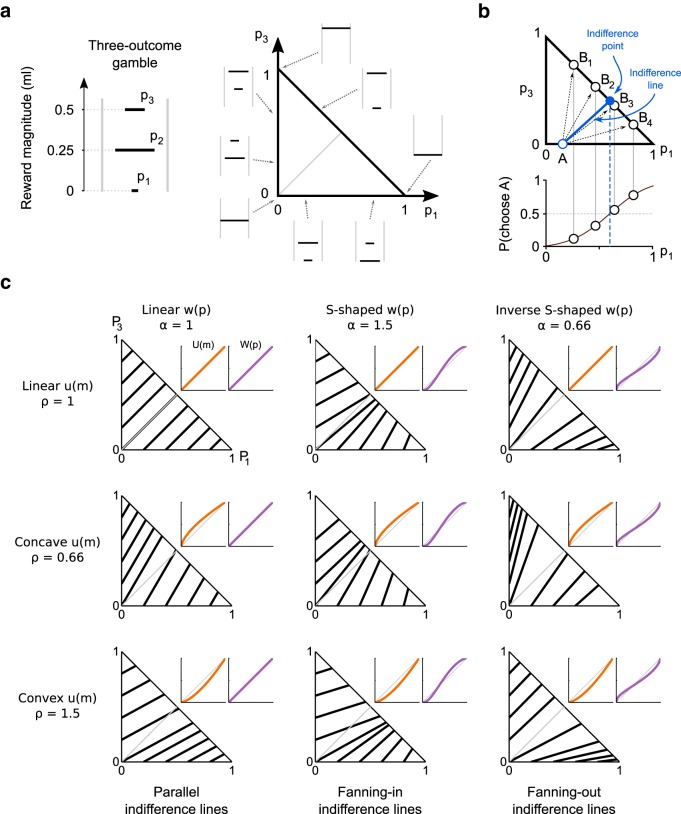
Indifference lines in the Marschak–Machina triangle modeling different patterns of probability distortion. ***a***, Representation of gambles in the Marschak–Machina triangle. Schematic representation of a three-outcome gamble (left): probabilistic combination (*p*_1_, *p*_2_, *p*_3_) of three fixed magnitudes (*m*_1_ = 0 ml, *m*_2_ = 0.25 ml, *m*_3_ = 0.50 ml), which can be represented in the Marschak–Machina triangle (right, with example gambles corresponding to points on the triangle edges). Gray line in triangle connects points with equal EV (0.25 ml). ***b***, Procedure for the psychometric measurement of one indifference line. An indifference point (top, blue dot) in choices between a fixed gamble A and different gambles B_i_ (circles) was defined as the point on the triangle hypotenuse for which a softmax function fitted on the ratio of A over B_i_ choices equated 0.5 (bottom). An indifference line was then constructed by connecting such indifference point on the hypotenuse to the fixed gamble A (blue line). ***c***, Theoretical indifference lines. Indifference lines predicted by cumulative prospect theory, for different underlying shapes of utility (*u(m*), power function) and probability distortion (*w(p*), *Prelec-1* function). Each plot represents the indifference lines corresponding to a particular combination of *u* and *w* shapes, represented by orange and purple lines, respectively. The shape of the utility function (linear in the first row of plots, concave and convex in the other two rows) changes the global orientation of the indifference lines, without affecting their fanning direction. On the contrary, a change in probability distortion shape corresponds to a change in the fanning direction of indifference lines: a linear probability distortion (first column) produces parallel indifference lines, whereas S-shaped (second column) and inverse S-shaped (third column) probability distortions correspond to indifference lines fanning-in and fanning-out, respectively, regardless of the utility function shape.

The use of this diagram, which makes it possible to represent a more general class of choice options (i.e., gambles with three fixed outcomes of varying probabilities) (Fig. 5*a*), allowed us to extend our results to a wider range of problems. We did this to test the robustness of the parametric modeling (out-of-sample test) and, most importantly, to investigate the effect of elicitation condition from a different perspective: by looking at the change in direction of indifference lines, which connected points of the triangle edges (specific two-outcome gambles) for which the animal expressed choice indifference (Fig. 5*b*), we could quantify the effects of elicitation condition that were specifically dependent on changes in probability distortion, and independent of changes in the shape of the utility function.

One of the theoretical consequences of probability distortions in the Marschak–Machina triangle is that indifference lines would not be parallel to each other, as in the case of linear probability weighting, but would instead fan-out or fan-in depending on the probability distortion (Fig. 5*c*): an inverse S-shaped probability distortion would induce fanning-out, whereas an S-shaped one would result in indifference lines fanning-in. Fanning-out would indeed correspond to an increase in the steepness of the indifference lines when shifting “probability mass” from worse to better outcomes. As steeper lines correlate with more risk-seeking behavior, fanning-out would imply an inverse S-shaped probability distortion. The opposite would happen with fanning-in indifference lines, then corresponding to an S-shaped probability distortion function (Camerer, 1989). Crucially, because the outcome magnitudes used in the Marschak–Machina triangle are fixed, the fanning direction is independent of the utility function and is therefore solely determined by the shape of the probability distortion. In that sense, any observed change in the fanning direction of the indifference lines with a change in elicitation sequence could only be due to a change in the probability weighting function (Fig. 5*c*).

**Figure 6.**
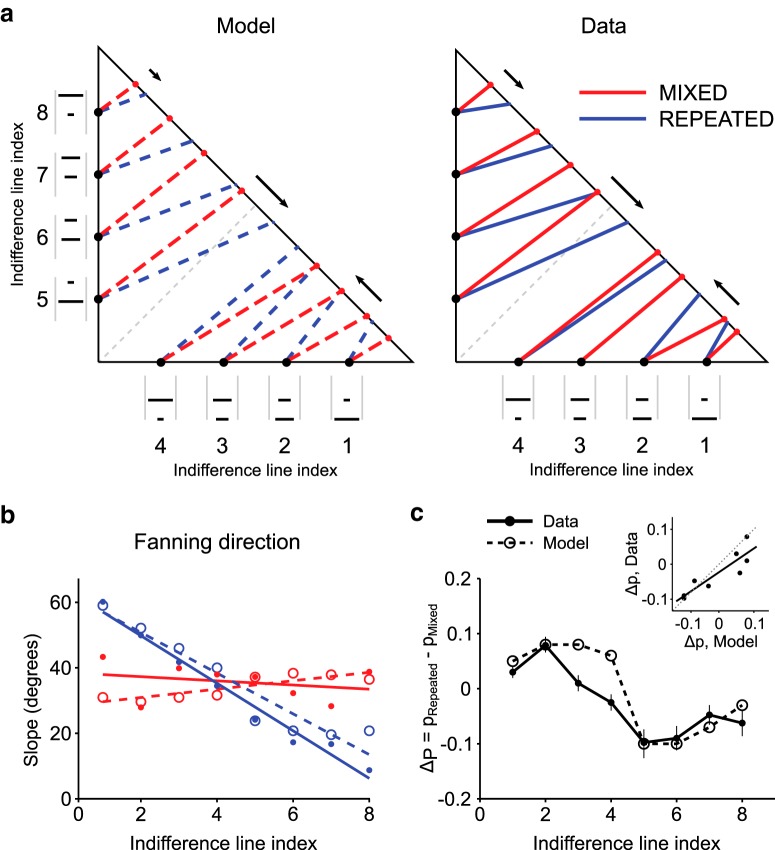
Effect of CE elicitation sequences on the Marschak–Machina triangle indifference lines. ***a***, Modeled (left) and measured (right) patterns of indifference lines across conditions. Arrows indicate the direction and amount of shift for three sample indifference points between the MIXED (red) and REPEATED (blue) conditions, highlighting how the model correctly predicted the effect of condition change. Gray line connects points with the same EV (0.25 ml), representing an indifference line in case of risk-neutral behavior. Numbers define indices for the indifference lines, corresponding to fixed gambles on the triangle edges (black dots, also represented as visual cues). ***b***, Fanning direction of the indifference lines. Points represent the slope of indifference lines (angle between each line and the horizontal axis) as a function of indifference line index. Circles represent the model predicted values. Dots represent experimental data. Lines indicate linear regressions, separately computed on the two task conditions for the model (dashed lines) and the data points (continuous lines). A regression line with negative slope corresponds to a decrease in indifference line angle, indicating fanning-out; conversely, a positive regression coefficient indicates fanning-in of indifference lines. ***c***, Statistical comparison between model and experimental data. Shift in location of indifference points across elicitation sequences (average difference ± SEM). A linear regression between the modeled and measured shifts (inset) confirmed the match between model and data in terms of predicted shift in indifference points, corresponding to a correct prediction of the change in the fanning direction across conditions.

We used the previously recovered parameters for utility and probability distortion to estimate the expected pattern of indifference lines in the two experimental conditions: MIXED and REPEATED sequences. We then compared the predicted directions of the indifference lines with the measured ones. As expected, the theoretical indifference lines, modeled using the previously elicited parameters, showed a slight fanning-out pattern for the MIXED condition, where a weakly inverse S-shaped probability distortion was measured. Conversely, we saw a fanning-in pattern in the REPEATED condition, for which we had observed an S-shaped probability distortion (Fig. 6*a*, left).

The direct experimental measure of indifference lines was performed by presenting the animals with binary choices between a gamble represented by a fixed point on the triangle edge and one of several points on the triangle's hypotenuse. The indifference line was defined as the segment connecting the fixed point with the point corresponding to choice indifference on the hypotenuse. This procedure resulted in a directional pattern of indifference lines compatible with the theoretically predicted one, with no clear fanning direction of indifference lines in the MIXED condition, and clear fanning-in in the REPEATED condition (Fig. 6*a*, right). We quantified this directional pattern of indifference lines using a measure for the fanning direction. The fanning of indifference lines corresponds to a gradual change in the slope of indifference lines: when moving from the lower right corner of the probability triangle to the upper left corner, an increasing slope would produce fanning-out, whereas a decreasing slope would produce fanning-in. Following this principle, we statistically assessed the fanning direction of the indifference lines by computing a linear regression on the slopes of the indifference lines. Results show no significant regression slope in the MIXED condition (*R*^2^ = 0.08, *p* = 0.50), suggesting no fanning of indifference curves, whereas in the REPEATED condition a significant linear regression (*R*^2^ = 0.98, *p* = 4.4 × 10^−6^) indicated fanning-out of the indifference lines. These results are consistent with predictions from the modeled indifference lines, which show a similar pattern of fanning directions (Fig. 6*b*).

We statistically compared the measured and predicted patterns of indifference lines by calculating the shift in the location of indifference points across conditions, corresponding to changes in the slope of indifference lines. A significant correlation between the predicted and measured shifts (Pearson's correlation coefficient *r* = 0.78, *p* = 4.0 × 10^−3^) confirmed that the experimental data complied with our theoretical predictions (Fig. 6*c*) and supported the finding that probability distortion drove the change in risk attitude between REPEATED and MIXED conditions.

### The effect of trial history on the probability distortion

Because the structure of elicitation sequences appeared to affect probability distortions specifically, we investigated whether the differences in choice behavior could be explained in relation to past experiences, or trial history. One key difference between elicitation sequences was the order of the probabilities presented on the screen. In the MIXED sequences, the monkeys were much more likely to have experienced different gambles in their immediate past than in trials from REPEATED sequences, where the same gamble was repeated numerous times. Consequently, while the range of probabilities, magnitudes, and safe outcomes was identical in both conditions, the variability of past gambles was significantly different between the two conditions (Fig. 1*d*,*e*).

**Figure 7.**
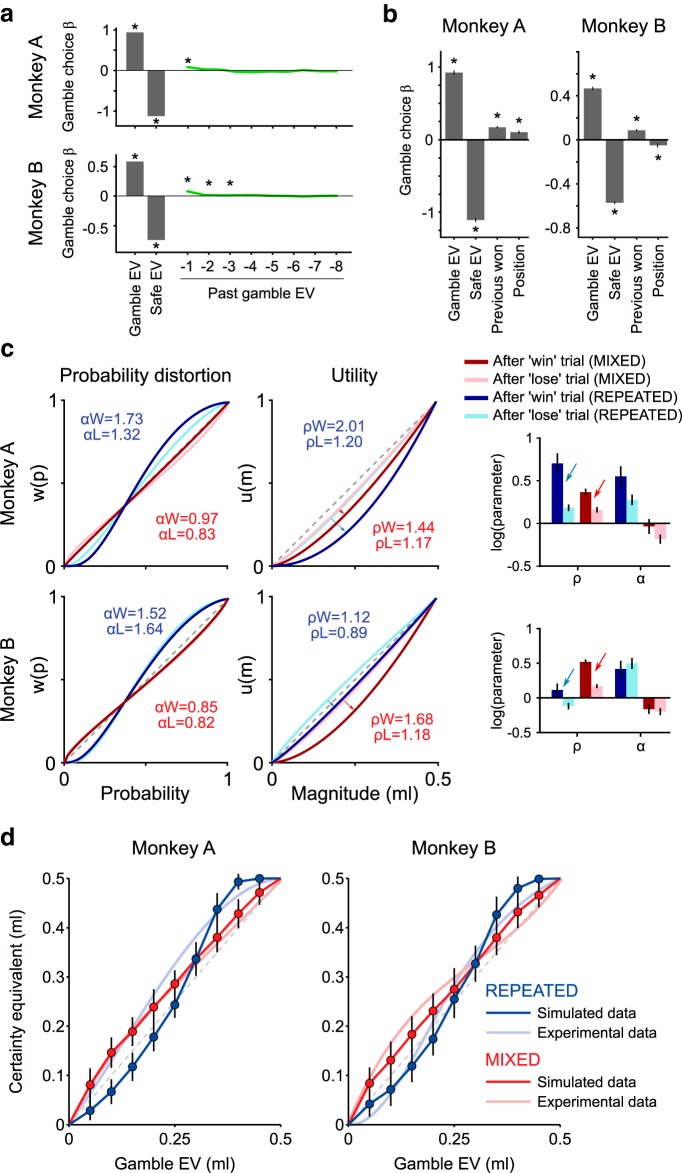
Sequence-dependent effects of trial history on probability distortion shape. ***a***, Influence of past trials on current trial's choice. Standardized regression coefficients (mean ± SEM across sessions) for current trial's gamble EV, safe reward magnitude, and previous trials' gamble EV (up to eight trials in the past). *Coefficients significantly different from zero (*t* test). For both monkeys, the choice behavior depended on at least one trial in the past. Positive regression coefficients indicated that an increase in the previous trial's gamble EV induced the monkeys to choose the current trial's gamble option more frequently. ***b***, Effect of the past outcomes on gamble choices. Standardized regression coefficients (mean ± SEM across sessions) for gamble EV, safe magnitude, previous trial's gamble outcome (0 or 0.5 ml), and gamble position. A significant positive coefficient for the previous outcome indicated that monkeys chose the gamble more often when the previously chosen gamble was successful (0.5 ml) than when it was not successful (0 ml): the gamble was chosen more after a win than after a loss. In terms of BIC score, this model (Eq. 15) was at least as good at describing the choice data compared with the model with no past trials' influence (Eq. 2) (Monkey A: BIC_2_ = 84.2, BIC_14_ = 82.3, *t* test: *p* = 0.14; Monkey B: BIC_2_ = 221.4, BIC_14_ = 215.8, *t* test: *p* = 5.8 × 10^−5^). ***c***, Effect of past outcomes on the utility and probability distortion functions. The utility function appeared more convex following a gamble-win trial (0.5 ml reward) than following a loss (no reward), suggesting that gamble outcomes had an influence on the relative value of gamble and safe options on the next trial. The utility parameter estimates following win and loss trials are indicated as αW and αL, respectively, whereas probability distortion parameter as ρW and ρL, respectively. Arrows indicate the change in the utility parameter between loss and win trials. Error bars indicate the 95% CIs of the parameter estimates. ***d***, Simulated effect of EV update mechanism based on past outcomes. Mean ± SEM across simulated sessions (*N* = 50) of the CE resulting from choices simulated using the learning model (Eq. 16) in MIXED and REPEATED conditions. The parameters used in the simulation were recovered from the MLE procedure with the same model separately for each monkey. Linear probability weighting and linear magnitude coding were used in the simulation, demonstrating that an EV update mechanism interacting with the local trial structure could explain the observed change in risk attitudes across conditions without explicitly introducing a nonlinear probability distortion.

Because human and nonhuman primates, much like rodents, often base part of their risky decisions on recent experiences (Nowak and Sigmund, 1993; Barron and Erev, 2003; Marshall and Kirkpatrick, 2013; Hayden et al., 2008), we again ran a logistic regression on the probability of choosing the gamble option: this time to verify whether the EV of past gambles had any impact on the animals' decisions (Eq. 14). We found that, in the MIXED condition, both monkeys made use of at least one past gamble to make their decision (Fig. 7*a*). The monkeys appeared to bias their choices in favor of the gamble (positive regression coefficient) when the prior gamble's EV was higher. In game-theoric terms, and taking the gamble's EV as a proxy for its “win rate,” monkeys seemed to follow a WSLS strategy, whereby receiving a reward from a risky choice option increased the likelihood of choosing a similar option again; the opposite was true for choices where the risky option resulted in a loss (no reward). To validate this hypothesis, we applied a WSLS-compatible model (Eq. 15) on the immediate trial history of both monkeys, looking at their propensity to choose a gamble over a safe outcome when they had previously chosen a gamble and won (Fig. 7*b*). As expected, we found a significant effect of both the current gamble's EV (one-sample *t* test, Monkey A: *t*_(50)_ = 29.41, *p* = 3.19 × 10^−33^; Monkey B: *t*_(58)_ = 32.28, *p* = 9.38 × 10^−39^) and the current safe outcome's EV on the likelihood of choosing a gamble (one-sample *t* test, Monkey A: *t*_(50)_ = −38.71, *p* = 6.05 × 10^−39^; Monkey B: *t*_(58)_ = −46.19, *p* = 1.9 × 10^−47^). Both monkeys had a small but significant side bias (one-sample *t* test, Monkey A: *t*_(50)_ = −4.59, *p* = 2.97 × 10^−5^; Monkey B: *t*_(58)_ = −2.55, *p* = 1.3 × 10^−2^). More importantly, there was a significant positive effect of “winning” the preceding gamble on the likelihood of selecting the gamble option again, regardless of value (one-sample *t* test, Monkey A: *t*_(50)_ = 10.75, *p* = 1.3 × 10^−14^; Monkey B: *t*_(58)_ = 8.32, *p* = 1.76 × 10^−11^). In other words, receiving a reward from a risky gamble made the next gamble more attractive relative to the safe outcome.

We investigated this effect further, by estimating separate utility and probability distortion parameters in trials where a past gamble had been selected and “won” and in trials where the past selected gamble had been “lost.” Due to lower trial counts per session after this trial selection, all sessions were pooled for each condition. In both animals, the utility function estimated from the former class of trials was more convex than the utility estimated from unrewarded trials (Fig. 7*c*). Probability distortions, however, were not consistently different between these two classes of trials, maintaining their respective inverse-S and S shapes for MIXED and REPEATED conditions. Much like in the logistic regression, these results suggested a tendency to choose the gamble option more often after rewarded (win) trials, compared with unrewarded trials (a more convex utility function corresponding to stronger risk-seeking behavior). What it also highlighted, however, was a change in the relative value distribution between gambles and safe options: one that varies with past experience. In other words, gambles following a rewarded trial would be of higher relative value for the monkeys than those following unrewarded trials, at least in terms of safe rewards.

Past win or lost effects on subjective value could account for some of the gap in probability distortion observed across our two conditions. A MIXED sequence of gambles would drive subjective value estimates in an opposing pattern to that of a REPEATED elicitation sequence simply due to task structure. In the case of MIXED sequences, the random distribution of gamble probabilities would indeed result in an inverse-S probability distortion. Gambles with probabilities >0.5 would, more often than not, follow a gamble of lower EV; the monkey would then, on average, be less likely to pick said gamble due to the decrease in subjective value estimate following lower past returns. This would drive down the CE value of high probability gambles. In the case of low probability gambles, high past returns would drive CEs up. From this, we would expect an opposing distortion pattern in a REPEATED condition. For any gamble, the CE value would be distorted in a way proportional to its own probability: a low probability gamble would be driven down in value by repeated experience, whereas a high probability gamble would see its value go up. A change in gamble value, rather than a simple WSLS strategy, might also have longer lasting effects and could explain the persistence of sequence-type effects when looking at choices in the Marschak–Machina triangle paradigm.

To test this hypothesis directly, we developed a simple reinforcement learning model in which gamble values were updated based on the previous trial's outcome: the value of a gamble increased by a fixed amount after a win and decreased by the same amount after a loss (Eq. 16). Importantly, in the choice model, the gambles' starting values were the respective objective EVs, which were compared with the objective safe magnitudes to make choices. No utility or probability distortion was included, only the previous choice softmax function, and we made no distinction between parameters estimated in repeated or mixed sequences. We again estimated the model parameters through MLE on each session's trial-by-trial choice data and retrieved a significant, mean value-updating parameter for both monkeys (Monkey A: η = 4.5 × 10^−3^ ± 9.0 × 10^−4^ SEM; *t*_(55)_ = 4.96, *p* = 7.1 × 10^−6^; Monkey B: η = 4.1 × 10^−3^ ± 5.8 × 10^−4^ SEM; *t*_(58)_ = 7.1, *p* = 2.0 × 10^−9^). The value of η corresponded to the fixed amount of value being added to or removed from the gamble's subjective value estimate following “win” and “lose” trials, respectively.

After running the estimation procedure on all sessions, we tested whether the average observed value-updating parameter could explain the different CE distributions seen across the MIXED and REPEATED conditions. We computed CEs from simulated choices using the learning model defined above (Eq. 16), using the mean recovered softmax and value-updating parameters, still holding utility and probability weights linear. The resulting pattern of simulated CEs (Fig. 7*d*) followed the experimental pattern. In particular, it captured the clear separation between the two CE elicitation sequences. Although this model appeared to have a higher BIC score than the “classical” prospect theory model (Eq. 3) (Monkey A: BIC_Eq16_ = 160.7, BIC_Eq3_ = 137.5, *t*_(55)_ = 6.92, *p* = 5.01 × 10^−9^; Monkey B: BIC_Eq16_ = 419.8, BIC_Eq3_ = 392.7, *t*_(58)_ = 4.69, *p* = 1.70 × 10^−5^), it accounted for the change in the pattern of CEs across both conditions using a single set of parameters. Conversely, two different sets of parameters were necessary for the prospect theory counterpart to capture the different CE patterns.

Together, these results suggest that a simple value-updating mechanism that modifies gamble values based on the previous outcomes, applied to different elicitation sequences, would be sufficient to induce a reversal in the observed probability distortion patterns of monkeys during choice.

## Discussion

This study demonstrated that the shape of the probability weighting function guiding value-based choices in monkeys depended largely on the task's sequence structure. When deriving CEs from sequences in which different probabilistic rewards pseudorandomly alternated (MIXED), we found that monkeys overweighted low probability rewards and underweighted high probability ones. Conversely, the same CE elicitation method yielded the opposite choice pattern (underweighting of low probabilities and overweighting of high ones) when choice sequences consisted of trial blocks each containing a unique, REPEATED gamble. By simultaneously eliciting utility and probability weighting functions from each of these elicitation conditions, we showed that the two opposing choice patterns we observed could be explained by a reversal of the standard inverse S-shaped probability distortion function, seen when gambles were MIXED, to an S-shaped distortion when identical gambles were REPEATED. We confirmed and extended these results by comparing choice indifference lines in the Marschak–Machina triangle representations of the two elicitation conditions. The triangle's indifference maps were compatible with the observed inversion of probability distortions, preserving the weighting patterns in trials where no safe options were presented. Finally, by analyzing both sequence structure and monkeys' choices in relation to previous trials, we showed that a past-driven update of subjective values could partially explain the observed reversal in probability distortion.

Modern economic theories of choice under risk introduced distorted probability weightings to account for biases and departures from expected utility theory's predictions (von Neumann and Morgenstern, 1944; Allais, 1953; Kahneman and Tversky, 1979). Since then, the typical finding has been that humans overweighted low probabilities, all the while underweighting high ones (Lattimore et al., 1992; Gonzalez and Wu, 1999; Abdellaoui, 2000; Tobler et al., 2008): an inverse-S probability distortion (Kahneman and Tversky, 1979). This shape has also been replicated in monkeys (Stauffer et al., 2015), where human-ported tasks resulted in a reliable inverse-S probability distortion. The current study ties in with these findings, using a coherent set of visual stimuli for both gambles and safe reward options to control for any bias introduced by the different visual representations of the two option types. Our results, in addition to reliability capturing macaque behavior using modern economic choice theories, further characterize the effects of sequence structure on utility and probability distortion.

In contrast to the generally reported inverse-S-shaped probability distortion, a growing number of studies on human and animal subjects have highlighted the variability in probability distortion shapes, both across subjects and between task conditions (Hey and Strazzera, 1989; Bruhin et al., 2010; Farashahi et al., 2018). Recent work by Farashahi et al. (2018) emphasized the flexibility of probability weights in adapting to contextual changes, after finding that S-shaped and linear probability distortions could be elicited in monkeys when performing different tasks. Our experimental data confirmed this high level of behavioral flexibility in monkeys, whereby directly manipulating the order of presented gambles in a single task produced opposing patterns of probability distortion.

Other findings from human experiments suggest that the way in which probability information is presented could account for the reported variability in subjects' risk attitudes. For example, when reward probabilities are explicitly described (choice from description) to human subjects, they act as if overweighting the probability of rare events, but when probabilities are learned from experience (choice from experience), subjects choose as if underweighting the probability of rare events. This effect has been aptly referred to as the description-experience (DE) gap (Hertwig et al., 2004) and appears to extend to other primates. Indeed, monkeys have been shown to be more risk-seeking for experienced than for described gambles, suggesting a similar DE gap effect in nonhuman primates (Heilbronner and Hayden, 2016). Whereas some authors have called for two separate theories explaining choices from description and choices from experience (Hertwig and Erev, 2009; Abdellaoui et al., 2011), others have suggested that prospect theory could effectively describe choice in the two situations when allowing for a change in the probability distortion function between the two settings (Ungemach et al., 2009; Frey et al., 2015).

While the dichotomous choice patterns we observed are comparable with those described in the DE gap studies, here the cues representing reward probabilities were identical in the two sequence conditions. In both MIXED and REPEATED sequences, probabilities were described explicitly through cues, learned from experience by the animals; the conditions only differed in the presentation order of the probability information. While the task design was different from previous human DE studies in this respect, the repeated sampling of outcomes typically used to “learn” the value of risky prospects in choices from experience (for review, see Wulff et al., 2018) echoes the repetitive structure of our REPEATED sequence; conversely, described prospects are typically presented in a less structured, randomized sequence, analogous to our MIXED condition. While a direct comparison remains to be made, findings in both the DE gap experiments and in the present study suggest that past trial outcomes play a role in shaping the subjective perception of reward probabilities.

Sampling bias has been identified as a source of variability in probability distortions, particularly in relation to the DE gap. Indeed, sampling bias is particularly problematic in “experienced” conditions due to the limited number of trials used in learning the options' values: with small sample sizes, low probability gambles are often rewarded less frequently than would be prescribed by their nominal probability (Hertwig and Erev, 2009; Hertwig and Pleskac, 2010; Camilleri and Newell, 2013). The use of identical descriptive cues and elicitation procedures in the present study ensured that similar sampling sizes were applied, and indeed required, to estimate CEs for every gamble. Any bias would therefore affect the two conditions in a similar manner. With no obvious sampling biases, our data suggest that the DE gap could be modeled on the probability distortion changes we observed across task conditions, and that much like in the present study, the observed changes in risk-preferences from described to experienced reward probabilities, might result from differences in the task's presentation order of probability information.

A final source of variability we considered was that the REPEATED condition was a much less complex decision situation than the MIXED one: one could ignore the gamble in long, repeated sequences. However, we found that the animals neither differentially weighed the options nor made choices faster in the REPEATED condition, indicating that they were not using widely differing valuation strategies.

The Marschak–Machina triangle, a diagram widely used in the economics literature, allows for the intuitive representation of choices between two- and three-outcome gambles, serving as an ideal framework for investigating complex economic choice problems (Machina, 1987; Camerer, 1989). In the current experiment, we elicited indifference points in the Marschak–Machina triangle representation of the monkeys' behavior, which crucially provided a link between animal and human studies. Although full indifference curves within the Marschak–Machina triangle remain to be tested, we showed that indifference points on the triangle edges complied with economic theories of choice, and confirmed the reversal of probability distortion across conditions, this time with probabilistic rewards only. Consequently, we demonstrated the possibility of rigorous behavioral characterization in nonhuman primates, paving the way for future investigations into the neurophysiological basis of advanced economic constructs, such as probability distortion, specific economic axioms, or the neural counterparts of alternative economic theories.

In conclusion, our results demonstrated the effect of a task's sequence structure on the shape of a monkey's elicited probability distortion, and highlighted the potential influence of past rewards on subjective value. Moreover, and perhaps most significantly, these adaptive effects extended through time: the patterns of indifference lines observed in the Marschak–Machina triangle after a session of MIXED or REPEATED sequences were compatible with the probability distortion shapes measured in the preceding CE elicitation session, even though the paradigm used in the Marschak–Machina triangle was always randomized. In this sense, the CE elicitation sequences preceding the Marschak–Machina triangle paradigm might have driven and reinforced a gap between the subjective values of identical probabilities, one that influenced choices between gambles in the Marschak–Machina triangle. The reinforcement learning model we used supports this hypothesis, implying that each experienced outcome could reinforce and update the subjective value of probabilities, leading to a flexible and contextually driven judgment of probabilistic information. More sophisticated models, such as the addition of a standard Rescorla–Wagner learning rule or a nonlinear transformation of safe magnitudes to the current value updating mechanism, could be more biologically plausible and successful in explaining the choice mechanism, and remain to be explored. It should be noted that the monkeys' initial learning/association phase was not analyzed here in reinforcement learning terms, as it was performed with imperative trials. A better understanding of probability learning, and the permanence of subjective values reinforced across different conditions, could shed light on the core elements of prospect theory and on the undeniably adaptive nature of utility and probability distortions.
