# Supplementary figures and images for: Probability Distortion Depends on Choice Sequence in Rhesus Monkeys
Source: J Neurosci. 2019 Apr 10;39(15):2915–29. doi: 10.1523/JNEUROSCI.1454-18.2018 (PMC6462447; doi:10.1523/JNEUROSCI.1454-18.2018)

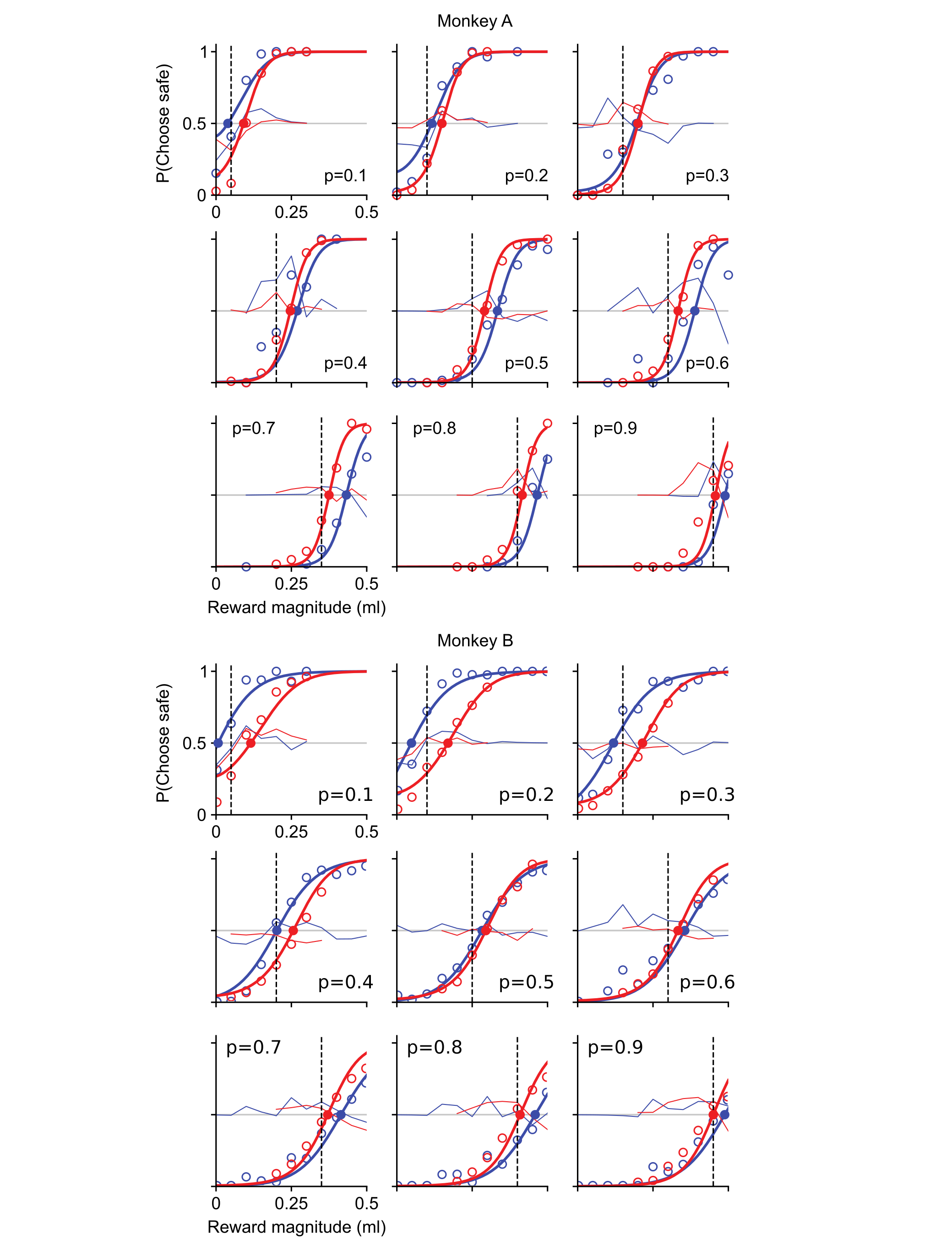

Supplement: Figure 2-1 [file sup_ns-JN-RM-1454-18-s01.tif]

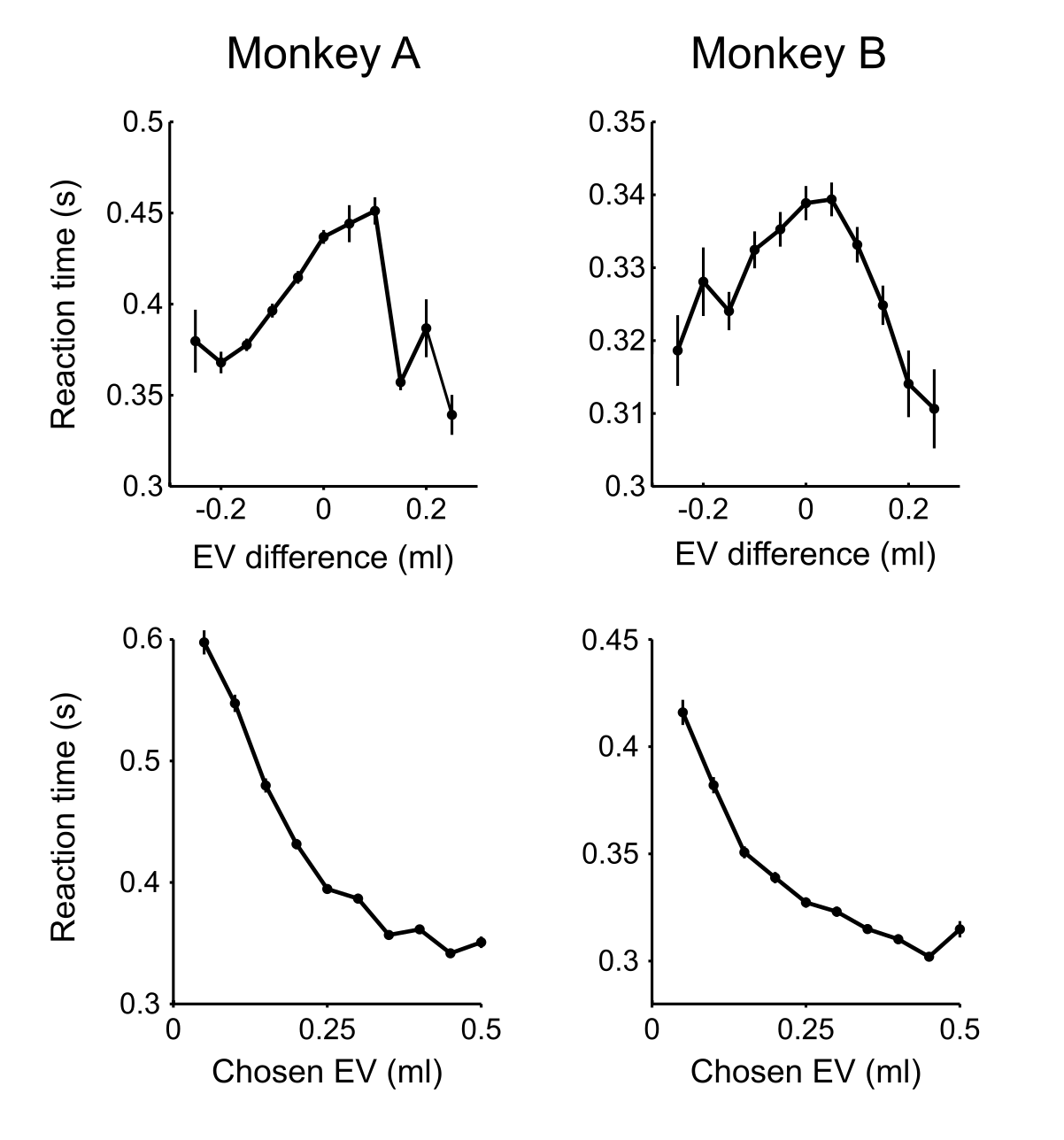

Supplement: Figure 3-1 [file sup_ns-JN-RM-1454-18-s02.tif]
